# Crystal structure of 5-(4-methyl­phen­yl)-3-[(*E*)-2-(4-methyl­phen­yl)ethen­yl]cyclo­hex-2-en-1-one

**DOI:** 10.1107/S2056989015008324

**Published:** 2015-05-07

**Authors:** Joel T. Mague, Shaaban K. Mohamed, Mehmet Akkurt, Antar A. Abdelhamid, Mustafa R. Albayati

**Affiliations:** a Department of Chemistry, Tulane University, New Orleans, LA 70118, USA; b Chemistry and Environmental Division, Manchester Metropolitan University, Manchester M1 5GD, England; c Chemistry Department, Faculty of Science, Minia University, 61519 El-Minia, Egypt; d Department of Physics, Faculty of Sciences, Erciyes University, 38039 Kayseri, Turkey; e Department of Chemistry, Faculty of Science, Sohag University, 82524 Sohag, Egypt; f Kirkuk University, College of Science, Department of Chemistry, Kirkuk, Iraq

**Keywords:** crystal structure, cyclo­hexenenones, α,β-unsaturated ketones, C—H⋯O inter­actions

## Abstract

In the title compound, C_22_H_22_O, the dihedral angle between the planes of the benzene rings is 53.55 (7)°. Weak C—H⋯O inter­actions help to direct the packing, forming sheets lying parallel to (020).

## Related literature   

For the synthesis of cyclo­hexenones and their use as synthons, see: Mayekar *et al.* (2010 ▸); Suwito *et al.* (2014 ▸); Tabba *et al.* (1995 ▸); Bella *et al.* (2012 ▸); Xing *et al.* (2010 ▸); Martin & Prasad (2006 ▸). For various biological activities of cyclo­hexenone derivatives, see: Prasad *et al.* (2006 ▸); Kumar *et al.* (2003 ▸); Tatsuzaki *et al. (*2006); Yun *et al.* (2006 ▸); Kim *et al.* (2008 ▸); Yoon *et al.* (2007 ▸); Tanaka *et al.* (1997 ▸); Vyas *et al.* (2009 ▸). For the use of cyclo­hexenones as inter­mediates in synthesis, see: Mayekar *et al.* (2010 ▸); Bella *et al.* (2012 ▸); Xing *et al. (*2010); Martin & Prasad (2006 ▸). For the bioactivity of dehydro­zingerone, chalcone and isoeugenol derivatives, see: Tatsuzaki *et al.* (2006 ▸).
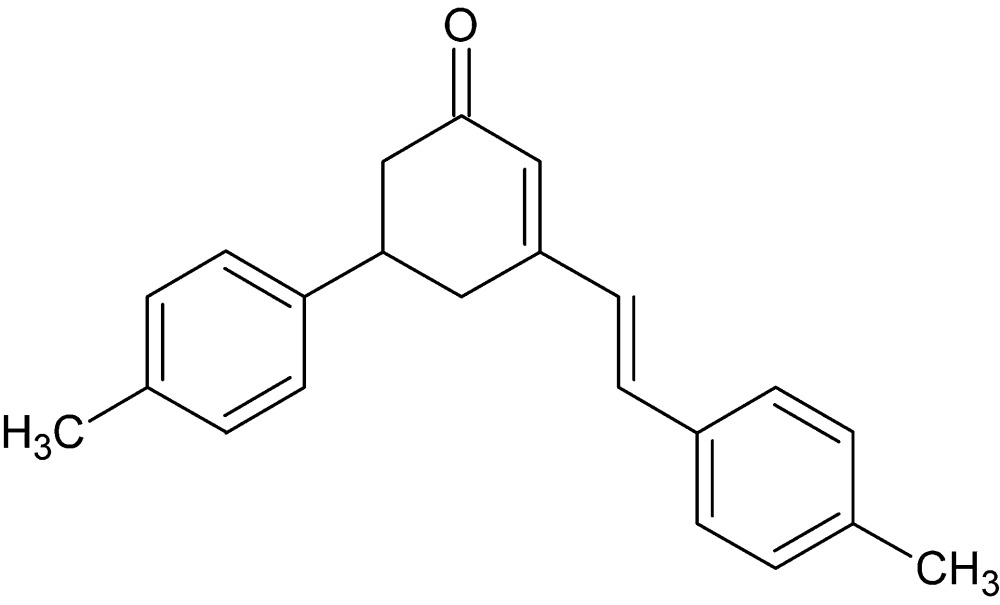


## Experimental   

### Crystal data  

C_22_H_22_O
*M* *_r_* = 302.39
Monoclinic, 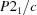

*a* = 4.9614 (1) Å
*b* = 30.7302 (6) Å
*c* = 11.0726 (2) Å
β = 93.268 (1)°
*V* = 1685.44 (6) Å^3^
*Z* = 4
Cu *K*α radiation
μ = 0.55 mm^−1^
*T* = 150 K
0.31 × 0.11 × 0.08 mm

### Data collection  

Bruker D8 VENTURE PHOTON 100 CMOS diffractometer
Absorption correction: multi-scan (*SADABS*; Bruker, 2014 ▸) *T*_min_ = 0.84, *T*_max_ = 0.96
12558 measured reflections
3247 independent reflections
2529 reflections with *I* > 2σ(*I*)
*R*_int_ = 0.042

### Refinement  

*R*[*F* ^2^ > 2σ(*F* ^2^)] = 0.050
*wR*(*F* ^2^) = 0.131
*S* = 1.05
3247 reflections
210 parameters
H-atom parameters constrained
Δρ_max_ = 0.35 e Å^−3^
Δρ_min_ = −0.19 e Å^−3^

### 

Data collection: *APEX2* (Bruker, 2014 ▸); cell refinement: *SAINT* (Bruker, 2014 ▸); data reduction: *SAINT*; program(s) used to solve structure: *SHELXT* (Sheldrick, 2015*a* ▸); program(s) used to refine structure: *SHELXL2014* (Sheldrick, 2015*b* ▸); molecular graphics: *DIAMOND* (Brandenburg & Putz, 2012 ▸); software used to prepare material for publication: *SHELXTL* (Sheldrick, 2008 ▸).

## Supplementary Material

Crystal structure: contains datablock(s) global, I. DOI: 10.1107/S2056989015008324/lr2135sup1.cif

Structure factors: contains datablock(s) I. DOI: 10.1107/S2056989015008324/lr2135Isup2.hkl

Supporting information file. DOI: 10.1107/S2056989015008324/lr2135Isup3.cml

**Fig. 1.**
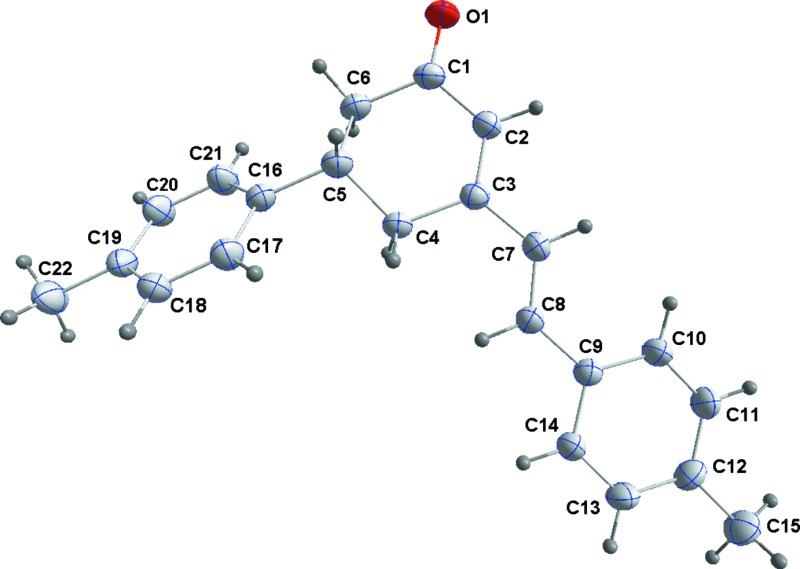
The title molecule with labeling scheme and 50% probability ellipsoids.

. DOI: 10.1107/S2056989015008324/lr2135fig1.tif
The title mol­ecule with labeling scheme and 50% probability ellipsoids.

**Fig. 2.**
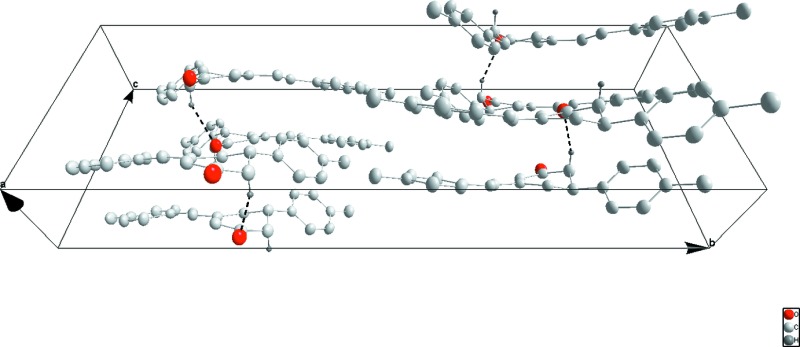
Packing viewed towards the (102)plane. Weak C—H···O interactions are shown as dotted lines.

. DOI: 10.1107/S2056989015008324/lr2135fig2.tif
Packing viewed towards the (10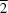)plane. Weak C—H⋯O inter­actions are shown as dotted lines.

CCDC reference: 1062089

Additional supporting information:  crystallographic information; 3D view; checkCIF report

## Figures and Tables

**Table 1:** Hydrogen-bond geometry (, )

*D*H*A*	*D*H	H*A*	*D* *A*	*D*H*A*
C6H6*A*O1^i^	0.99	2.60	3.515(2)	154
C8H8O1^ii^	0.95	2.47	3.353(2)	155
C14H14O1^ii^	0.95	2.55	3.410(2)	151

Symmetry codes: (i) 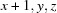; (ii) 
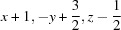
.

## supplementary crystallographic information

### S1. Structural commentary

From a chemical point of view, the most commonly used method for preparation of polyfunctionalized cyclo­hexenones is the Michael addition of carbanions to α,β-unsaturated ketones in presence of basic catalysts (Mayekar *et al*., 2010; Suwito *et al.*, 2014; Tabba *et al.*, 1995). Cyclo­hexenones have been considered as efficient synthons in building spiranic compounds (Mayekar *et al.*, 2010) or inter­mediates in the synthesis of fused heterocycles such as benzoselena­diazo­les and benzo­thia­zoles (Bella *et al*., 2012), benzo­pyrazoles (Xing *et al.*, 2010) or carbazole derivatives (Martin & Prasad, 2006). The existence of the α,β-unsaturated ketone moiety is a common feature of a large number of biologically active compounds which exhibit diverse pharmacological effects such as anti-microbial (Prasad *et al.*, 2006), anti-tumor (Kumar *et al.*, 2003), anti-cancer (Tatsuzaki *et al.*, 2006; Yun *et al.*, 2006) and radical scavenger activities (Kim *et al.*, 2008) as well as being inhibitors of topoisomerase I (Yoon *et al.*, 2007). Cyclo­hexenone derivatives, in particular, are well known lead molecules for the treatment of inflammation and autoimmune diseases (Tanaka *et al.*, 1997). Several reports have pointed out the importance of cyclo­hexenones for anti-microbial and anti-tubercular activity (Vyas *et al.*, 2009).

In the title compound (Fig. 1), the dihedral angle between the phenyl rings is 53.55 (7)°. Weak C6—H6A···O1^i^ (i: *x* + 1, *y*, *z*) inter­actions help to direct the packing (Fig. 2 and Table 1).

### S2. Synthesis and crystallization

In 30 ml of methanol, a mixture of 1 mmol (262 mg) of (1*Z*,4E)-1,5-bis­(4-methyl­phenyl)­penta-1,4-dien-3-one and 1 mmol (100 mg) of acetyl­acetone was refluxed for 5 h in the presence of 10 mg of sodium methoxide. The resulting solid product was collected, filtered under vacuum, washed with cold ethanol and recrystallized from ethanol to afford colourless columns which were suitable for X-ray diffraction. Mp. 371 K.

### S3. Refinement

Crystal data, data collection and structure refinement details are summarized in Table 1. H-atoms were placed in calculated positions (C—H = 0.95 - 0.98 Å) and included as riding contributions with isotropic displacement parameters 1.2 - 1.5 times those of the attached carbon atoms. The 020 reflection was omitted from the final refinement as it was partially obscured by the beamstop.

### Crystal data

**Table:**

C_22_H_22_O	*F*(000) = 648
*M**_r_* = 302.39	*D*_x_ = 1.192 Mg m^−^^3^
Monoclinic, *P*2_1_/*c*	Cu *K*α radiation, λ = 1.54178 Å
*a* = 4.9614 (1) Å	Cell parameters from 7504 reflections
*b* = 30.7302 (6) Å	θ = 2.9–72.6°
*c* = 11.0726 (2) Å	µ = 0.55 mm^−^^1^
β = 93.268 (1)°	*T* = 150 K
*V* = 1685.44 (6) Å^3^	Column, colourless
*Z* = 4	0.31 × 0.11 × 0.08 mm

### Data collection

**Table:**

Bruker D8 VENTURE PHOTON 100 CMOS diffractometer	3247 independent reflections
Radiation source: INCOATEC IµS micro-focus source	2529 reflections with *I* > 2σ(*I*)
Mirror monochromator	*R*_int_ = 0.042
Detector resolution: 10.4167 pixels mm^-1^	θ_max_ = 72.4°, θ_min_ = 4.3°
ω scans	*h* = −5→6
Absorption correction: multi-scan (*SADABS*; Bruker, 2014)	*k* = −38→36
*T*_min_ = 0.84, *T*_max_ = 0.96	*l* = −11→13
12558 measured reflections	

### Refinement

**Table:**

Refinement on *F*^2^	Primary atom site location: structure-invariant direct methods
Least-squares matrix: full	Secondary atom site location: difference Fourier map
*R*[*F*^2^ > 2σ(*F*^2^)] = 0.050	Hydrogen site location: inferred from neighbouring sites
*wR*(*F*^2^) = 0.131	H-atom parameters constrained
*S* = 1.05	*w* = 1/[σ^2^(*F*_o_^2^) + (0.0579*P*)^2^ + 0.6292*P*] where *P* = (*F*_o_^2^ + 2*F*_c_^2^)/3
3247 reflections	(Δ/σ)_max_ = 0.001
210 parameters	Δρ_max_ = 0.35 e Å^−^^3^
0 restraints	Δρ_min_ = −0.19 e Å^−^^3^

### Special details

**Table:**

Geometry. All e.s.d.'s (except the e.s.d. in the dihedral angle between two l.s. planes)are estimated using the full covariance matrix. The cell e.s.d.'s are takeninto account individually in the estimation of e.s.d.'s in distances, anglesand torsion angles; correlations between e.s.d.'s in cell parameters are onlyused when they are defined by crystal symmetry. An approximate (isotropic)treatment of cell e.s.d.'s is used for estimating e.s.d.'s involving l.s. planes.
Refinement. Refinement of *F*^2^ against ALL reflections. The weighted *R*-factor *wR* and goodness of fit *S* are based on *F*^2^, conventional *R*-factors *R* are based on *F*, with *F* set to zero for negative *F*^2^. The threshold expression of *F*^2^ > σ(*F*^2^) is used only for calculating *R*-factors(gt) *etc*. and is not relevant to the choice of reflections for refinement. *R*-factors based on *F*^2^ are statistically about twice as large as those based on *F*, and *R*- factors based on ALL data will be even larger. H-atoms were placed in calculated positions (C—H = 0.95 - 0.98 Å) and included as riding contributions with isotropic displacement parameters 1.2 - 1.5 times those of the attached carbon atoms.

### Fractional atomic coordinates and isotropic or equivalent isotropic displacement parameters (Å^2^)

**Table:**

	*x*	*y*	*z*	*U*_iso_*/*U*_eq_	
O1	−0.2370 (3)	0.78680 (4)	0.50574 (12)	0.0422 (3)	
C1	−0.0733 (4)	0.77848 (6)	0.42988 (16)	0.0324 (4)	
C2	−0.0376 (4)	0.73445 (6)	0.38590 (16)	0.0320 (4)	
H2	−0.1435	0.7119	0.4177	0.038*	
C3	0.1391 (3)	0.72401 (5)	0.30189 (15)	0.0280 (4)	
C4	0.2939 (4)	0.75888 (5)	0.23953 (15)	0.0298 (4)	
H4A	0.2923	0.7521	0.1521	0.036*	
H4B	0.4841	0.7583	0.2718	0.036*	
C5	0.1810 (4)	0.80493 (6)	0.25518 (16)	0.0331 (4)	
H5	0.0083	0.8068	0.2043	0.040*	
C6	0.1127 (4)	0.81281 (6)	0.38459 (17)	0.0369 (4)	
H6A	0.2814	0.8132	0.4368	0.044*	
H6B	0.0257	0.8417	0.3905	0.044*	
C7	0.1933 (4)	0.67865 (6)	0.27681 (16)	0.0313 (4)	
H7	0.0800	0.6575	0.3107	0.038*	
C8	0.3907 (4)	0.66425 (5)	0.20954 (15)	0.0295 (4)	
H8	0.4884	0.6858	0.1686	0.035*	
C9	0.4725 (3)	0.61914 (6)	0.19209 (15)	0.0296 (4)	
C10	0.3664 (4)	0.58378 (6)	0.25307 (18)	0.0389 (4)	
H10	0.2265	0.5885	0.3067	0.047*	
C11	0.4619 (4)	0.54214 (6)	0.23644 (18)	0.0392 (4)	
H11	0.3877	0.5188	0.2800	0.047*	
C12	0.6632 (4)	0.53343 (6)	0.15784 (17)	0.0355 (4)	
C13	0.7678 (4)	0.56848 (6)	0.09696 (17)	0.0394 (5)	
H13	0.9057	0.5635	0.0424	0.047*	
C14	0.6762 (4)	0.61052 (6)	0.11375 (16)	0.0341 (4)	
H14	0.7534	0.6338	0.0712	0.041*	
C15	0.7656 (5)	0.48793 (6)	0.1393 (2)	0.0466 (5)	
H15A	0.7277	0.4700	0.2095	0.070*	
H15B	0.9608	0.4888	0.1300	0.070*	
H15C	0.6750	0.4754	0.0664	0.070*	
C16	0.3727 (4)	0.83857 (5)	0.20647 (15)	0.0310 (4)	
C17	0.4337 (4)	0.83682 (6)	0.08492 (16)	0.0348 (4)	
H17	0.3555	0.8146	0.0345	0.042*	
C18	0.6061 (4)	0.86683 (6)	0.03656 (16)	0.0357 (4)	
H18	0.6431	0.8648	−0.0465	0.043*	
C19	0.7260 (4)	0.89973 (6)	0.10667 (17)	0.0339 (4)	
C20	0.6674 (4)	0.90135 (6)	0.22736 (18)	0.0394 (4)	
H20	0.7471	0.9234	0.2777	0.047*	
C21	0.4943 (4)	0.87140 (6)	0.27656 (17)	0.0378 (4)	
H21	0.4585	0.8734	0.3597	0.045*	
C22	0.9152 (4)	0.93214 (7)	0.0529 (2)	0.0450 (5)	
H22A	1.1003	0.9209	0.0606	0.068*	
H22B	0.9060	0.9599	0.0960	0.068*	
H22C	0.8619	0.9366	−0.0328	0.068*	

### Atomic displacement parameters (Å^2^)

**Table:**

	*U*^11^	*U*^22^	*U*^33^	*U*^12^	*U*^13^	*U*^23^
O1	0.0419 (8)	0.0429 (7)	0.0440 (8)	0.0040 (6)	0.0210 (6)	−0.0051 (6)
C1	0.0284 (9)	0.0378 (10)	0.0315 (9)	0.0061 (7)	0.0061 (7)	0.0004 (7)
C2	0.0292 (9)	0.0332 (9)	0.0344 (9)	0.0010 (7)	0.0078 (7)	0.0028 (7)
C3	0.0261 (8)	0.0302 (9)	0.0277 (9)	0.0019 (7)	0.0017 (6)	0.0011 (6)
C4	0.0315 (9)	0.0296 (9)	0.0287 (9)	0.0011 (7)	0.0067 (7)	−0.0015 (6)
C5	0.0362 (9)	0.0314 (9)	0.0323 (9)	0.0015 (7)	0.0074 (7)	−0.0021 (7)
C6	0.0390 (10)	0.0312 (9)	0.0417 (10)	0.0040 (8)	0.0116 (8)	−0.0033 (7)
C7	0.0325 (9)	0.0292 (9)	0.0329 (9)	−0.0021 (7)	0.0076 (7)	0.0017 (7)
C8	0.0340 (9)	0.0285 (8)	0.0263 (8)	−0.0009 (7)	0.0051 (7)	0.0002 (6)
C9	0.0326 (9)	0.0289 (9)	0.0276 (9)	−0.0004 (7)	0.0038 (7)	−0.0006 (6)
C10	0.0439 (11)	0.0328 (9)	0.0417 (10)	−0.0010 (8)	0.0176 (8)	−0.0004 (8)
C11	0.0471 (11)	0.0299 (9)	0.0415 (11)	−0.0027 (8)	0.0109 (8)	0.0032 (7)
C12	0.0420 (10)	0.0287 (9)	0.0354 (10)	0.0038 (8)	−0.0006 (8)	−0.0027 (7)
C13	0.0438 (11)	0.0358 (10)	0.0401 (10)	0.0053 (8)	0.0150 (8)	−0.0029 (8)
C14	0.0402 (10)	0.0306 (9)	0.0326 (9)	−0.0001 (7)	0.0115 (8)	0.0010 (7)
C15	0.0545 (13)	0.0334 (10)	0.0522 (13)	0.0081 (9)	0.0064 (10)	−0.0006 (9)
C16	0.0358 (9)	0.0267 (8)	0.0309 (9)	0.0027 (7)	0.0069 (7)	−0.0011 (7)
C17	0.0418 (10)	0.0309 (9)	0.0321 (9)	−0.0019 (8)	0.0058 (7)	−0.0044 (7)
C18	0.0418 (10)	0.0350 (10)	0.0312 (9)	0.0033 (8)	0.0102 (8)	0.0012 (7)
C19	0.0322 (9)	0.0305 (9)	0.0396 (10)	0.0032 (7)	0.0077 (7)	0.0023 (7)
C20	0.0435 (11)	0.0352 (10)	0.0399 (11)	−0.0067 (8)	0.0064 (8)	−0.0068 (8)
C21	0.0468 (11)	0.0361 (10)	0.0315 (10)	−0.0041 (8)	0.0099 (8)	−0.0056 (7)
C22	0.0444 (12)	0.0414 (11)	0.0506 (12)	−0.0057 (9)	0.0143 (9)	0.0013 (9)

### Geometric parameters (Å, º)

**Table:**

O1—C1	1.228 (2)	C11—H11	0.9500
C1—C2	1.452 (2)	C12—C13	1.387 (3)
C1—C6	1.506 (3)	C12—C15	1.506 (2)
C2—C3	1.352 (2)	C13—C14	1.386 (2)
C2—H2	0.9500	C13—H13	0.9500
C3—C7	1.450 (2)	C14—H14	0.9500
C3—C4	1.508 (2)	C15—H15A	0.9800
C4—C5	1.535 (2)	C15—H15B	0.9800
C4—H4A	0.9900	C15—H15C	0.9800
C4—H4B	0.9900	C16—C21	1.390 (3)
C5—C6	1.511 (2)	C16—C17	1.397 (2)
C5—C16	1.524 (2)	C17—C18	1.386 (3)
C5—H5	1.0000	C17—H17	0.9500
C6—H6A	0.9900	C18—C19	1.388 (3)
C6—H6B	0.9900	C18—H18	0.9500
C7—C8	1.339 (2)	C19—C20	1.385 (3)
C7—H7	0.9500	C19—C22	1.514 (3)
C8—C9	1.460 (2)	C20—C21	1.391 (3)
C8—H8	0.9500	C20—H20	0.9500
C9—C14	1.394 (2)	C21—H21	0.9500
C9—C10	1.398 (2)	C22—H22A	0.9800
C10—C11	1.381 (3)	C22—H22B	0.9800
C10—H10	0.9500	C22—H22C	0.9800
C11—C12	1.388 (3)		
			
O1—C1—C2	121.44 (16)	C12—C11—H11	119.1
O1—C1—C6	121.55 (16)	C13—C12—C11	117.25 (16)
C2—C1—C6	116.89 (15)	C13—C12—C15	121.09 (17)
C3—C2—C1	123.22 (16)	C11—C12—C15	121.66 (17)
C3—C2—H2	118.4	C14—C13—C12	121.60 (17)
C1—C2—H2	118.4	C14—C13—H13	119.2
C2—C3—C7	119.64 (15)	C12—C13—H13	119.2
C2—C3—C4	120.88 (15)	C13—C14—C9	121.04 (16)
C7—C3—C4	119.37 (14)	C13—C14—H14	119.5
C3—C4—C5	113.86 (14)	C9—C14—H14	119.5
C3—C4—H4A	108.8	C12—C15—H15A	109.5
C5—C4—H4A	108.8	C12—C15—H15B	109.5
C3—C4—H4B	108.8	H15A—C15—H15B	109.5
C5—C4—H4B	108.8	C12—C15—H15C	109.5
H4A—C4—H4B	107.7	H15A—C15—H15C	109.5
C6—C5—C16	113.91 (15)	H15B—C15—H15C	109.5
C6—C5—C4	110.97 (14)	C21—C16—C17	117.07 (16)
C16—C5—C4	110.25 (14)	C21—C16—C5	123.69 (16)
C6—C5—H5	107.1	C17—C16—C5	119.24 (16)
C16—C5—H5	107.1	C18—C17—C16	121.24 (17)
C4—C5—H5	107.1	C18—C17—H17	119.4
C1—C6—C5	112.23 (15)	C16—C17—H17	119.4
C1—C6—H6A	109.2	C17—C18—C19	121.53 (17)
C5—C6—H6A	109.2	C17—C18—H18	119.2
C1—C6—H6B	109.2	C19—C18—H18	119.2
C5—C6—H6B	109.2	C20—C19—C18	117.33 (17)
H6A—C6—H6B	107.9	C20—C19—C22	121.69 (17)
C8—C7—C3	125.02 (16)	C18—C19—C22	120.98 (17)
C8—C7—H7	117.5	C19—C20—C21	121.51 (17)
C3—C7—H7	117.5	C19—C20—H20	119.2
C7—C8—C9	127.22 (16)	C21—C20—H20	119.2
C7—C8—H8	116.4	C16—C21—C20	121.32 (17)
C9—C8—H8	116.4	C16—C21—H21	119.3
C14—C9—C10	117.34 (16)	C20—C21—H21	119.3
C14—C9—C8	118.65 (15)	C19—C22—H22A	109.5
C10—C9—C8	123.97 (16)	C19—C22—H22B	109.5
C11—C10—C9	120.96 (17)	H22A—C22—H22B	109.5
C11—C10—H10	119.5	C19—C22—H22C	109.5
C9—C10—H10	119.5	H22A—C22—H22C	109.5
C10—C11—C12	121.80 (17)	H22B—C22—H22C	109.5
C10—C11—H11	119.1		
			
O1—C1—C2—C3	179.54 (18)	C10—C11—C12—C15	−179.6 (2)
C6—C1—C2—C3	−4.5 (3)	C11—C12—C13—C14	0.1 (3)
C1—C2—C3—C7	170.34 (16)	C15—C12—C13—C14	−179.66 (19)
C1—C2—C3—C4	−5.8 (3)	C12—C13—C14—C9	−0.6 (3)
C2—C3—C4—C5	−14.9 (2)	C10—C9—C14—C13	0.3 (3)
C7—C3—C4—C5	169.05 (15)	C8—C9—C14—C13	177.83 (17)
C3—C4—C5—C6	44.1 (2)	C6—C5—C16—C21	5.5 (3)
C3—C4—C5—C16	171.31 (15)	C4—C5—C16—C21	−120.04 (19)
O1—C1—C6—C5	−149.12 (18)	C6—C5—C16—C17	−174.85 (17)
C2—C1—C6—C5	34.9 (2)	C4—C5—C16—C17	59.6 (2)
C16—C5—C6—C1	−179.03 (15)	C21—C16—C17—C18	−0.6 (3)
C4—C5—C6—C1	−53.9 (2)	C5—C16—C17—C18	179.74 (17)
C2—C3—C7—C8	−169.72 (18)	C16—C17—C18—C19	0.2 (3)
C4—C3—C7—C8	6.4 (3)	C17—C18—C19—C20	0.2 (3)
C3—C7—C8—C9	172.86 (17)	C17—C18—C19—C22	179.64 (18)
C7—C8—C9—C14	177.15 (18)	C18—C19—C20—C21	−0.3 (3)
C7—C8—C9—C10	−5.5 (3)	C22—C19—C20—C21	−179.72 (19)
C14—C9—C10—C11	0.4 (3)	C17—C16—C21—C20	0.5 (3)
C8—C9—C10—C11	−176.95 (18)	C5—C16—C21—C20	−179.83 (18)
C9—C10—C11—C12	−0.9 (3)	C19—C20—C21—C16	0.0 (3)
C10—C11—C12—C13	0.6 (3)		

### Hydrogen-bond geometry (Å, º)

**Table:**

*D*—H···*A*	*D*—H	H···*A*	*D*···*A*	*D*—H···*A*
C6—H6*A*···O1^i^	0.99	2.60	3.515 (2)	154
C8—H8···O1^ii^	0.95	2.47	3.353 (2)	155
C14—H14···O1^ii^	0.95	2.55	3.410 (2)	151

Symmetry codes: (i) *x*+1, *y*, *z*; (ii) *x*+1, −*y*+3/2, *z*−1/2.
