# Supplementary figures and images for: Crystal structure of 5-(4-methyl­phen­yl)-3-[(E)-2-(4-methyl­phen­yl)ethen­yl]cyclo­hex-2-en-1-one
Source: Acta Crystallogr E Crystallogr Commun. 2015 May 7;71(Pt 6):o436–7. doi: 10.1107/S2056989015008324 (PMC4459340; doi:10.1107/S2056989015008324)

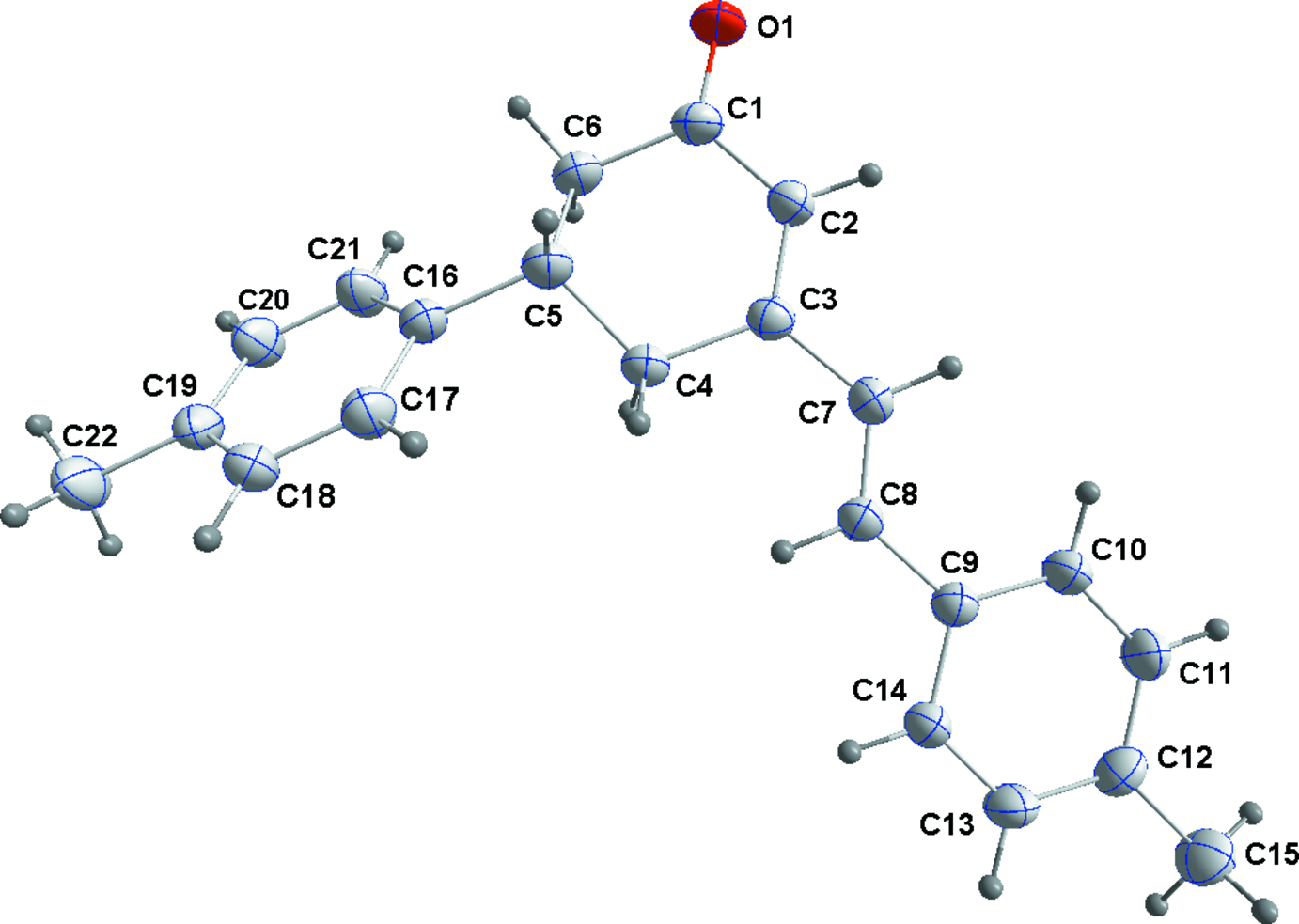

Supplement: Supplementary file 4 [file e-71-0o436-fig1.tif]

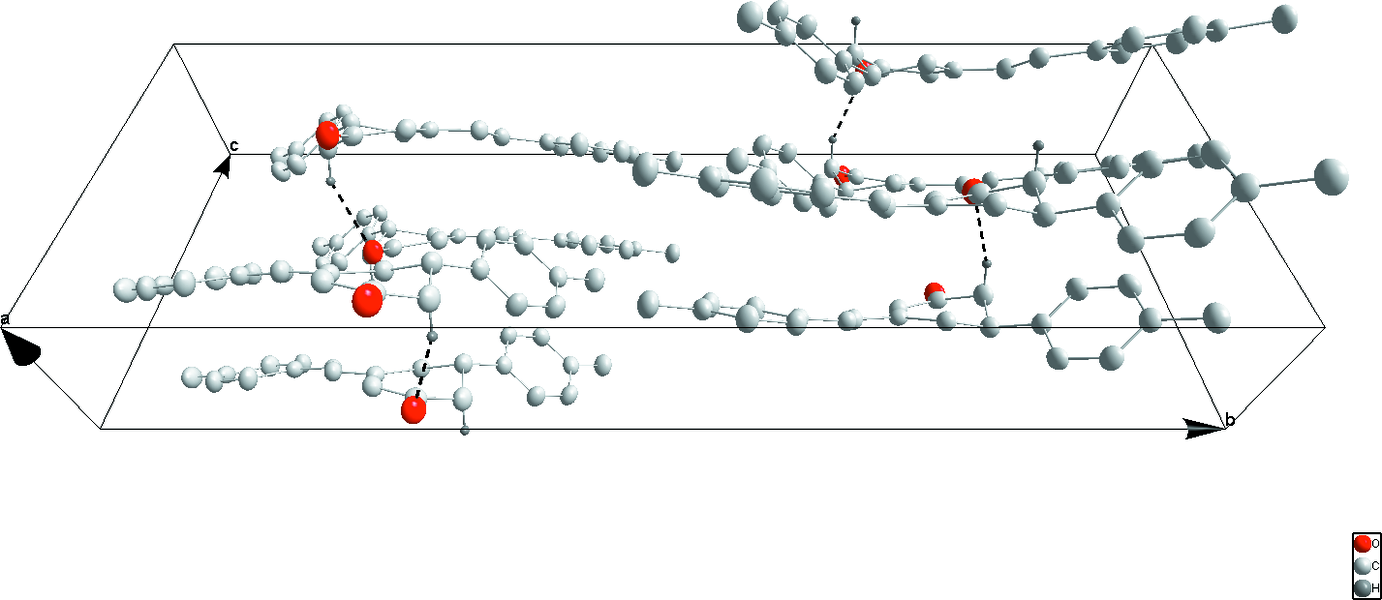

Supplement: Supplementary file 5 [file e-71-0o436-fig2.tif]
